# Drug repurposing to tackle parainfluenza 3 based on multi-similarities and network proximity analysis

**DOI:** 10.3389/fphar.2024.1428925

**Published:** 2024-10-01

**Authors:** Xinyue Chen, Bo Zhou, Xinyi Jiang, Huayu Zhong, Aijing You, Taiyan Zou, Chengcheng Zhou, Xiaoxiao Liu, Yonghong Zhang

**Affiliations:** ^1^ Chongqing Key Research Laboratory for Drug Metabolism, College of Pharmacy, Chongqing Medical University, Chongqing, China; ^2^ Department of Pharmacy, Children’s Hospital of Chongqing Medical University, Chongqing, China; ^3^ The Second Clinical College of Chongqing Medical University, Chongqing, China; ^4^ Medical Data Science Academy, College of Medical Informatics, Chongqing Medical University, Chongqing, China; ^5^ Chongqing Engineering Research Center for Clinical Big-Data and Drug Evaluation, Chongqing Medical University, Chongqing, China

**Keywords:** parainfluenza 3, drug repurposing, chemical similarity, disease similarity, network proximity

## Abstract

Given that there is currently no clinically approved drug or vaccine for parainfluenza 3 (PIV3), we applied a drug repurposing method based on disease similarity and chemical similarity to screen 2,585 clinically approved chemical drugs using PIV3 potential drugs BCX-2798 and zanamivir as our controls. Twelve candidate drugs were obtained after being screened with good disease similarity and chemical similarity (*S* > 0.50, *T* > 0.56). When docking them with the PIV3 target protein, hemagglutinin-neuraminidase (HN), only oseltamivir was docked with a better score than BCX-2798, which indicates that oseltamivir has an inhibitory effect on PIV3. After the distance ( $Z_{dc}$ ) between the drug target of 14 drugs and the PIV3 disease target was measured by the network proximity method based on the PIV3 disease module, it was found that the $Z_{dc}$ values of amikacin, oseltamivir, ribavirin, and streptomycin were less than those of the control. Thus, oseltamivir is the best potential drug because it met all the above screening requirements. Additionally, to explore whether oseltamivir binds to HN stably, molecular dynamics simulation of the binding of oseltamivir to HN was carried out, and the results showed that the *RMSD* value of the complex tended to be stable within 100 ns, and the binding free energy of the complex was low (−10.60 kcal/mol). It was proved that oseltamivir screened by our drug repurposing method had the potential feasibility of treating PIV3.

## Introduction

There is no specific antiviral treatment for parainfluenza (PIV) illness. Most people with PIV illness will recover on their own. However, PIVs can also cause more serious illnesses in children and adults older than 65 years, including bronchitis, bronchiolitis, and pneumonia (Moscona, 2005; Schmidt et al., 2011). Among PIVs, parainfluenza 3 (PIV3) is the most prevalent subtype, and it is not only a most common cause of acute respiratory infections in infants, but it also causes severe respiratory symptoms in older adults, immunocompromised patients, and transplant recipients (Branche and Falsey, 2016). Despite causing serious health problems, there is currently no clinically approved drug or vaccine for PIV3 (Contreras et al., 2021; Rafeek et al., 2021). PIV3 infects its target cells through the coordinated action of the hemagglutinin-neuraminidase receptor-binding protein (HN) and the fusion envelope glycoprotein, which together comprise the molecular fusion machinery (Palmer et al., 2014; Van Den Bergh et al., 2022). Peptide-fusion protein inhibitors that target the fusion envelope glycoprotein are challenging to utilize in clinical settings because of limitations such as high manufacturing costs, low oral bioavailability, and severe injection site reactions caused by the immunogenicity of virus-derived peptides (Aggarwal and Plemper, 2020). Current preclinical development and clinical trials for treating PIV3 primarily focus on inhibitors that target the HN protein (Chibanga et al., 2019; Rota et al., 2023).

At present, the common PIV3 drug research and development is carried out on HN inhibitors using virtual screening by molecular docking. Molecular docking is one of the most commonly used strategies in structure-based drug design (Parihar et al., 2022; Tayubi and Madar, 2023) and has been widely employed in the development of anti-PIV3 drugs (Indumathi et al., 2019; Bhasin et al., 2022). Zanamivir successfully docked with HN, and it has been proven to have anti-PIV3 effects *in vitro*, but is not suitable for clinical use due to its relatively high IC50 (Bailly et al., 2016). In the current clinical studies of PIV3, BCX-2798 has been shown to inhibit HN in mouse models, and this research has only recently progressed to the clinical stage. Further trials are necessary before it can be practically applied (Alymova et al., 2005). However, these classical screening methods often ignore the progression of the disease, and drug safety also needs to be fully assessed.

Recently, drug repurposing strategies, with the advantage of drug safety, have become strong approaches to the research and development of antiviral drugs (Kumar et al., 2021; Sonkar et al., 2021). The disease similarity and chemical similarity were calculated to uncover associations between diseases and drugs for use in drug repurposing (Li et al., 2018; Yin et al., 2023; Pushpakom et al., 2019). Interestingly, zanamivir and BCX-2798 are both derived from Neu5Ac2en, the neuraminidase (NA) inhibitor (Alymova et al., 2004). Their ability to inhibit PIV3 suggests that structurally similar compounds often share similar physicochemical properties and biological activities (Durai et al., 2020). Apart from structure-based virtual screening (Khan et al., 2023), the network-based drug-disease proximity method that uncovers the relationship between drug targets and disease modules is widely used (Yildirim et al., 2007; Zhou et al., 2020), and it is utilized not only for predicting the potential side effects but also for repurposing approved drugs for new indications (Guney et al., 2016; Wang et al., 2021; Xi et al., 2023).

Therefore, this research focuses on the following: 1) multi-similarity methods to understand the intrinsic link between diseases and drugs, 2) the structural characteristic link of potential drugs, and 3) the network proximity of disease targets and drug targets to understand drugs related to the occurrence and onset of diseases. We combined disease and chemical similarity methods to screen candidate drugs against PIV3. Molecular docking and network proximity methods were used to identify the best anti-PIV3 drug. Finally, the binding ability and stability of candidate drugs and key disease targets in a dynamic environment were evaluated using molecular dynamics simulation.

## Methods

**FIGURE 1:**
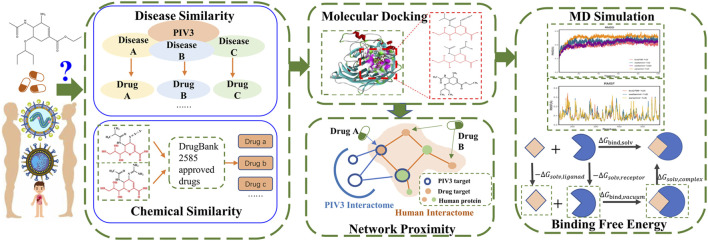
Workflow of PIV3 drug repurposing.

Our approach involves collecting data, calculating disease similarity to PIV3, and assessing chemical similarity to potential existing drugs for PIV3. We further screen through molecular docking and assess network proximity between the drug and PIV3. Finally, we conduct molecular dynamics simulations and calculate the binding free energy. The workflow is shown in Figure 1.

### Data collection

The Medical Subject Headings (MeSH) for PIV3 were obtained from https://nlmpubs.nlm.nih.gov/projects/mesh/2022/meshtrees/ and were used to calculate the disease similarity of PIV to all other diseases in the MeSH database that contain Medical Subject Headings. We obtained the approved drugs (molecular weight <500) from the DrugBank database (https://go.drugbank.com) as candidate drugs for drug repurposing. Zanamivir and BCX-2798 (potential therapeutic drugs for PIV3) were considered two controls.

Disease targets associated with PIV3 were obtained by using “*parainfluenza*”as a search term from GeneCards (https://www.genecards.org/), OMIM (https://www.omim.org/), and DisGeNET (https://www.disgenet.org/), excluding non-coding RNA proteins. The drug targets were sourced from the DrugBank database and DGIdb (https://dgidb.org). All data were downloaded on 26 March 2024. Disease genes and drug targets were sorted out with the same format in UniProt using Python 3.9.

### Multi-similarity analysis

Multi-similarity analysis, including disease therapy similarity and drug chemical similarity, was used to virtually screen all candidate drugs. The disease therapy similarity is calculated based on the directed acyclic graph (*DAG*), which is constructed using grid descriptors (Wang et al., 2007). The disease PIV3 are represented by ${DAG}_{\left(H\right)}=\left\{T_{H},E_{H}\right\}$ , where $T_{H}$ is the set of ancestor nodes containing node PIV3 and ${\;E}_{H}$ represents the set of edges from the parent node to the child node. In ${DAG}_{\left(H\right)}$ , the contribution of a certain node N to PIV3 ( ${SV}_{H}\left(n\right)$ ) is calculated as Equation 1:

$${SV}_{H}\left(n\right)=\left\{\begin{array}{l} 1\;if\;n=H\\ \max\left\{∆*{SV}_{H}\left(n^{\prime }\right)|n^{\prime }\epsilon children\;of\;n\right\}\;if\;n\neq H \end{array}\right.$$ (1)

The semantic similarity of PIV3 and disease A ( $S_{\left(H,A\right)}$ ) is calculated by Equation 2. If $S_{\left(H,A\right)}$ >0.5, it indicates that there is a good similarity between these two diseases.

$$S_{\left(H,A\right)}=\frac{\sum_{n\in N\left(H\right)\cap N\left(A\right)}\left({SV}_{H}\left(n\right)+{SV}_{H}\left(A\right)\right)}{SV\left(H\right)+SV\left(A\right)}$$ (2)

The Tanimoto coefficient (*T*) was calculated to assess the chemical similarity of all drugs. After the MACCS fingerprints of all drugs described by PaDEL (Yap, 2011), *T* is calculated using Equation 3:

$$T=\frac{c}{a+b-c}$$ (3)

where *a* and *b* are the numbers of MACCS fingerprint bits of drug A and B, respectively, and *c* is the number of fingerprint bits that two drugs have the same value on the MACCS fingerprint bits. If one drug has *bigger T*, the drug is a potential candidate.

The drugs obtained through the multi-similarity method are the initial candidates for the next round of screening.

### Molecular docking

After the candidates were screened by multi-similarity analysis, we employed molecular docking to assess the docking and interaction between the screened drugs and the key target HN (PDB ID: 1V2I) (Indumathi et al., 2019). We utilized a literature-based method to identify the binding sites due to the absence of binding ligands in the original PDB structure. The key amino acids ARG192, ASP216, GLU409, ARG424, ARG502, TYR530, and GLU549 were designated as active sites (Lawrence et al., 2004; Mizuta et al., 2014; Bhasin et al., 2022). Molecular docking analyses were conducted on the selected candidates and two controls with HN using Discovery Studio 2019 (DS 2019).

### Network proximity analysis

Meanwhile, the network proximity calculation will be performed to assess the shortest path lengths between the candidate drug targets and PIV3 disease module (Wang et al., 2022; Tang et al., 2023). This evaluation will determine if the candidate drugs have the potential to treat PIV3.

PIV3 disease targets were distributed throughout the protein–protein-interaction (PPI) network (Menche et al., 2015; Dai et al., 2020), forming the PIV3 disease module and constructing the largest connected component (LCC) within the PIV3 disease module. The shortest path length (*d*_*c*_, Equation 4) of the PIV3 disease target set (V) and drug target set (T) is as follows:

$$d_{c}\left(V,T\right)=\frac{1}{\left|T\right|}\sum_{t\in T}\min_{v\in V}d\left(v,t\right)$$ (4)

Using the average ${\;\mu }_{d}\left(V,T\right)$ and standard deviation $\sigma_{d}\left(V,T\right)$ of the reference distribution between randomly selected protein groups, the calculated distance is converted into the average relative distance between drugs and PIV3 diseases. Equation 5 is for calculating the relative average shortest distance between V and T. If $Z_{dc}$ <0, which means that the distance between the test drug and the PIV3 disease module is less than the value of the reference distance, then the test drug is a potential candidate for anti-PIV3 (Morselli Gysi et al., 2021).

$${\;Z}_{dc}=\frac{dc-\mu_{dc}\left(V,T\right)}{\sigma_{dc}\left(V,T\right)}$$ (5)

### Molecular dynamics simulation

After completing the aforementioned screening methods, we identified drugs that met all the screening requirements and conducted molecular dynamics (MD) simulations of the drugs and HN complexes using GROMACS 2022.3.

Our simulation utilized the CHARMM36 force field and TIP3P solvent (Nayar et al., 2011), with the addition of a 0.15 mol/L NaCl solution to mimic physiological conditions. The steepest descent method was chosen for energy minimization (Koulgi et al., 2021). To replicate a physiological environment, a Langevin thermostat with a pressure of 1 atm and a temperature of 310 K was applied. The particle mesh Ewald (PME) algorithm was employed for calculating long-range interactions (Darden et al., 1993; Gu et al., 2011). Moreover, the simulation employed a step size of 2 fs/step, with a total of 50,000,000 steps, resulting in a simulating duration of 100 ns. Post-simulation, an analysis was conducted on the root-mean-square deviation (*RMSD*), root-mean-square fluctuation (*RMSF*), and radius of gyration (*RG*) of the complex.

Additionally, the binding free energy for each frame trajectory in the last 10 ns (90–100 ns) of the MD simulation was computed using the MMPBSA.py script and the MMGBSA.py (Valdés-Tresanco et al., 2021). The total binding energy is denoted as Δ*G*, and the Equations 6–8 for calculating Δ*G* are as follows:

$$∆\text{Gsol}=∆G_{NP}+∆G_{P}$$ (6)

$$∆\text{EMM}=∆G_{vdw}+∆G_{ele}+∆G_{\mathrm{int}}$$ (7)

∆G TOTAL=∆G sol+∆EMM (8)

where ∆Gsol represents the solvation free energy and ∆EMM represents the gas phase energy. ∆EMM is also equal to the sum of van der Waals energy ( $∆G_{vdw}$ ), internal energy ( $∆G_{\mathrm{int}}$ ), and electrostatic energy ( $∆G_{ele}$ ), whereas ∆Gsol is equal to the sum of polar solvation free energy ( $∆G_{P}$ ) and non-polar solvation free energy ( $∆G_{NP}$ ).

## Results

### Data collection

In this research, 2,585 approved drugs were obtained from the DrugBank database (see Supplementary Table S1A) as candidates. Subsequently, the chemical similarity between the controls (zanamivir and BCX-2798) and the 2,585 candidates were calculated, respectively. The results are listed in Supplementary Table S1B, C. At the same time, 324 PIV3 targets were compiled from the GeneCards, OMIM, and DisGeNET databases (see Supplementary Table S2).

### Multi-similarity analysis

First, we calculated the semantic similarity of 1,364 diseases to PIV3 (see Supplementary Table S3). Only three diseases were identified with an *S* > 0.5 to PIV3, including respiratory syncytial virus (RSV) (*S* = 0.78), Ebola virus (*S* = 0.55), and Orthomyxoviridae (*S* = 0.51). Till now, to treat these three diseases, there were only four clinically approved drugs, which are zanamivir, oseltamivir, ribavirin, and peramivir.

After the chemical similarity of 2,585 candidates to the two controls were calculated, it was found that only 12 drugs had *T* > 0.50, as shown in Table 1 and Supplementary Figure S1. It means that only 12 drugs had higher structure similarity to the controls. From Table 1, the chemical similarity between zanamivir and BCX-2798 is significant (*T* = 0.77). When we observed their molecular structures, in Supplementary Figure S1, it was noticed that they share the same pyranic acid core, polyhydroxy side chain, and amide side chain. Similar to the control, most of the screened drugs contain oxygen-containing hexatomic rings, carboxyl groups, and amide structures. These functional groups may serve as crucial structures for inhibiting the activity of PIV3 and warrant further exploration.

**TABLE 1:** Results of drug repurposing.

No.	Drug	*T* (zanamivir)	*T* (BCX-2798)	*CE* (kcal/mol)	*Z*_*dc*_
1	Streptomycin	0.67	0.56	−19.26	−0.95
2	Acarbose	0.64	0.60	—	−0.11
3	Cytarabine	0.64	0.56	−7.59	2.40
4	Streptozocin	0.63	0.72	−19.26	−0.69
5	Amikacin	0.63	0.59	—	−2.47
6	Fosdenopterin	0.63	0.56	−22.88	1.21
7	N-acetylglucosamine	0.61	0.57	−21.62	0.80
8	Oseltamivir	0.61	0.57	−24.69	−1.45
9	Plazomicin	0.61	0.59	—	−0.27
10	Peramivir	0.61	0.50	−18.90	—
11	Telbivudine	0.60	0.56	−8.05	—
12	Riboflavin	0.59	0.56	−14.98	0.12
13	Regadenoson	0.57	0.56	10.01	1.21
14	Ribavirin	0.54	0.57	10.85	**−2.27**
^a^	Zanamivir	^a^1.00	0.77	−31.10	−0.93
^a^	BCX-2798	0.77	^a^1.00	−23.05	—

^a^ Represents the control, and *CE* is CDOCKER energy calculated by DS.

Therefore, because two of the four candidates belonged to the 12, there were 14 selected candidates based on disease similarity (*S*_*(H,A)*_ >0.5) and chemical similarity (*T* > 0.56) (as shown in Table 1).

### Drug candidates docking to HN

**FIGURE 2:**
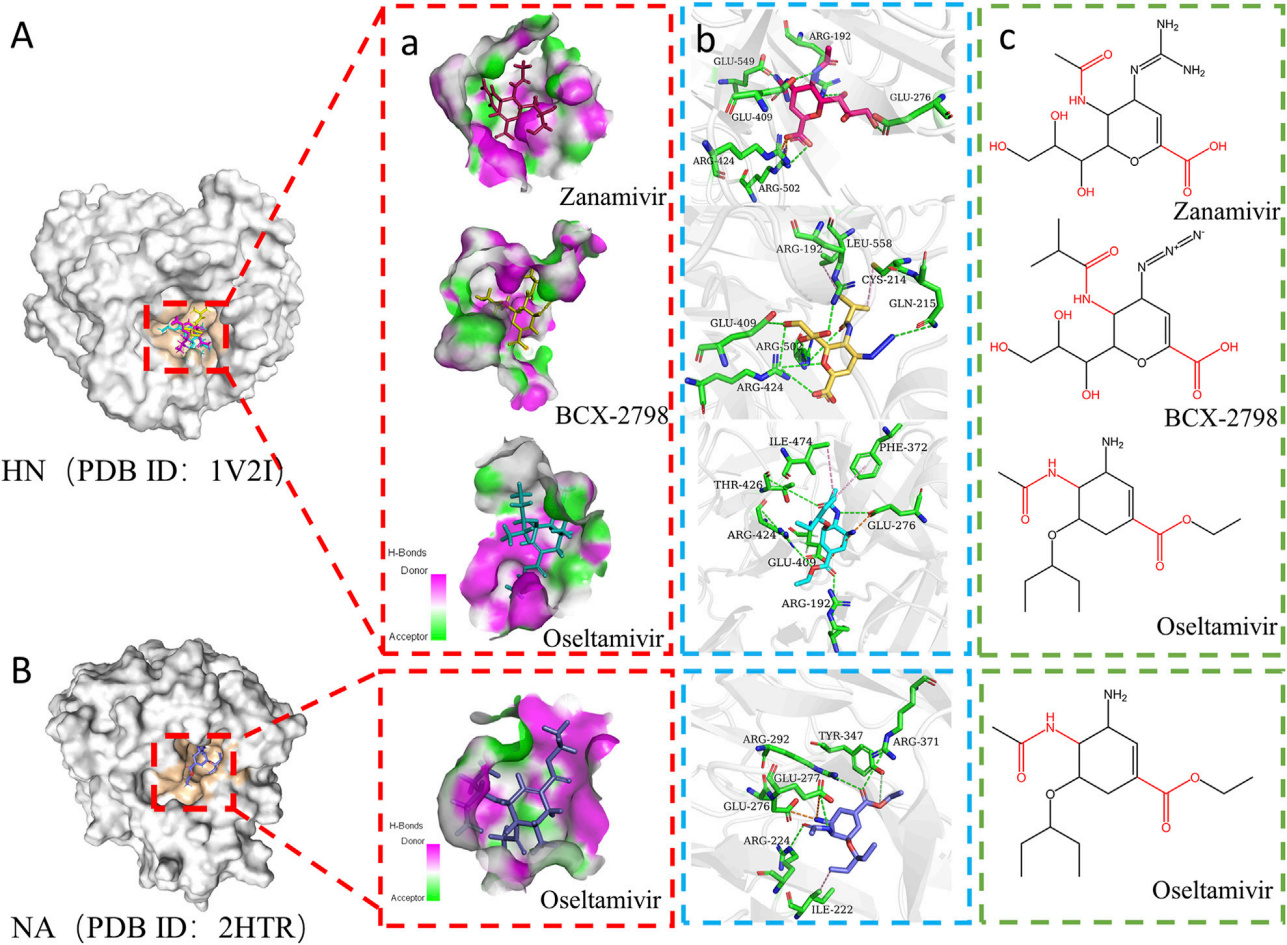
Interaction modes of the three drugs docked to different proteins. **(A)** Interaction diagram of three small molecules with HN (PDB ID:1V2I). **a)** Pocket diagram of zanamivir, BCX-2798, and oseltamivir at the same binding site. Pink areas represent amino acids in pockets as hydrogen bond donors. The green area represents the amino acid in the pocket as the hydrogen bond acceptor. The red stick is zanamivir, the yellow stick is BCX-2798, and the blue stick is oseltamivir. **b)** Interaction between three small molecules and key amino acid residues in binding pockets. The green stick is the key amino acid residue. The green dotted line represents hydrogen bonds, the orange dotted line represents electrostatic attraction, and the pink dotted line represents hydrophobic interaction. **c)** Chemical structure diagram of three molecules. **(B)** Interaction diagram of oseltamivir with NA (PDB ID:2HTR).

The 14 candidates were molecularly docked with HN (PDB ID: 1V2I), and their docking energy values are listed in Table 1. It was found that only oseltamivir (*CE* = −24.69 kal/mol) had a lower docking energy value than BCX-2798 (*CE* = −23.05 kal/mol), which indicated that oseltamivir bound to HN more stably than BCX-2798. Among all complexes, the top three drugs with stronger interactions were zanamivir, oseltamivir, and BCX-2798, so oseltamivir was the best among all the candidates. Visualization was carried out using DS 2019 and PyMol 2.5.0, as illustrated in Figure 2. We observed that all three compounds were stably bound to the pocket groove. Zanamivir was located in the shallow part of the binding pocket, revealing good flexibility and more options for binding poses. It was the same for BCX-2798. Oseltamivir occupied the deeper areas of the binding pocket, showing that oseltamivir had low flexibility, but it formed a more tightly bound complex. Oseltamivir was a good potential inhibitor of HN.

**FIGURE 3:**
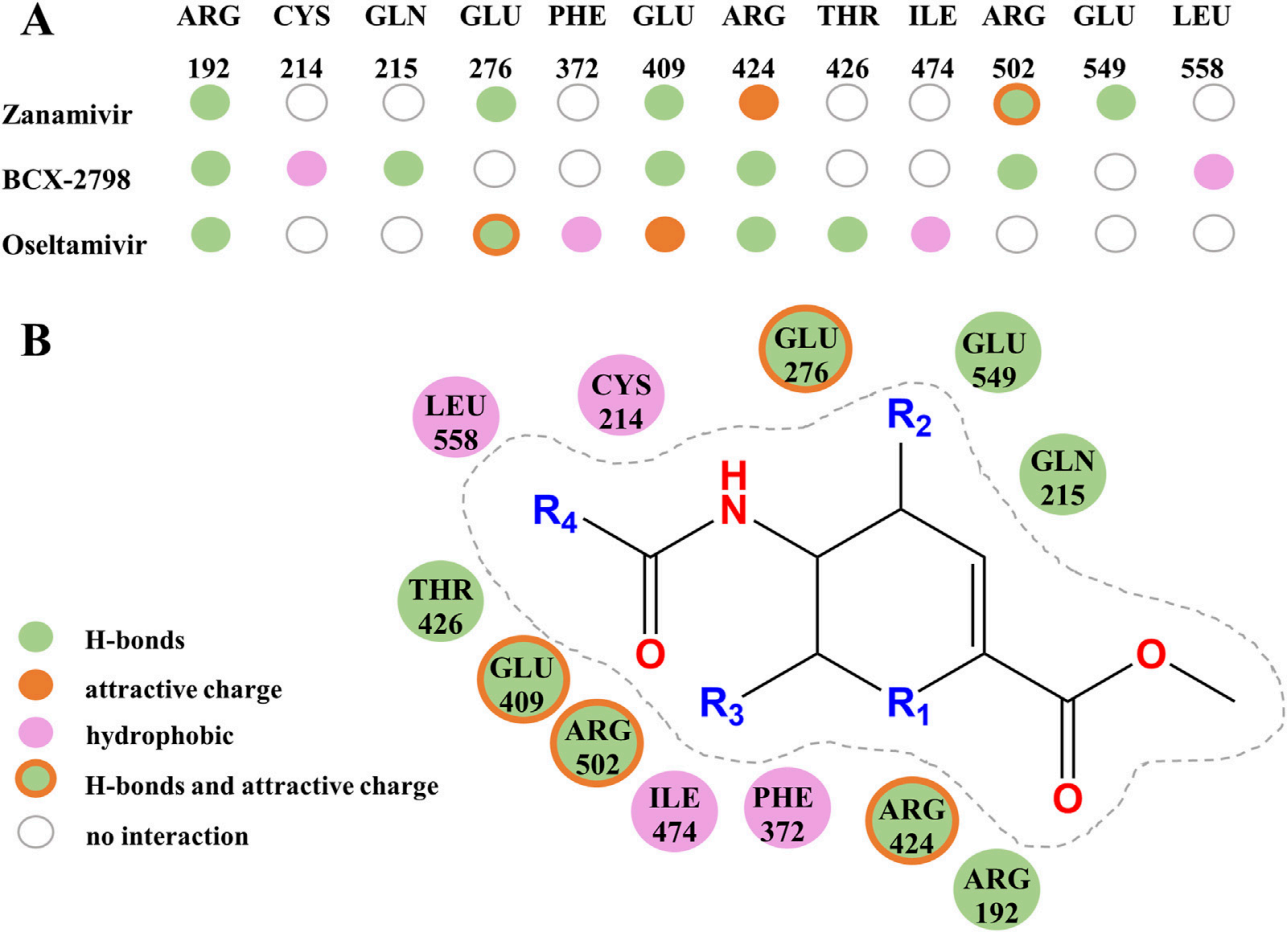
Amino residues and structural contributions of three drugs’ docking with HN. **(A)** Key amino acids combine with three kinds of small molecules to produce various interactive contributions. **(B)** Contribution of important chemical structures and groups to the formation of various interactions.

Furthermore, the intermolecular interactions of three complexes were observed and analyzed, and the contribution of key amino acid residues in binding pockets was explored Figures 2A, 3A. As shown in Figure 2A, in the three complexes, there were hydrogen bonding, electrostatic attraction, hydrophobic interaction, and some other intermolecular forces between small-molecule compounds and key amino acid residues, which promoted the stability of intermolecular binding. Most of the hydrogen bonds and electrostatic attraction in the oseltamivir–HN complex were contributed by ARG and GLU residues, similar to the two control–HN complexes. Especially, the amino of oseltamivir was bound to GLU276, amide was bound to THR426, and carboxyl groups were bound to ARG192 and ARG424 of HN. Compared with the controls, since the alkylation of oseltamivir increased the hydrophobicity of the carboxyl side chain, it can be found that there were some Pi–alkyl interactions with ILE474 and PHE372 in oseltamivir, leading to more stable binding. Therefore, it can be concluded that oseltamivir has a strong bond–bond interaction with amino acids around the HN protein-binding pocket.

Oseltamivir was a successful classical drug designed case when it was considered a neuraminidase (NA) enzyme inhibitor in influenza viruses (Davies, 2010). Then, we compared the molecular docking results of oseltamivir and NA (PDB ID: 2HTR) (Russell et al., 2006) to explore the interaction between oseltamivir and PIV3. As shown in Figure 2B, the side-chain amides, carboxyl groups, and amino groups of oseltamivir played crucial roles in inhibiting HN, similar to inhibiting NA (Laborda et al., 2016). Particularly, the acetamido fragment was well-accommodated within the cavity-binding domain both in HN and NA (Guillon et al., 2014). Thus, oseltamivir has the potential to inhibit PIV3.

Based on the contribution of amino residues binding to the pocket in Figure 3A and the common parent nucleus of three compounds (zanamivir, oseltamivir, and BCX-2798), the structural formula of potential lead compounds is derived, as shown in Figure 3B. From Figure 3B, the main structural differences among the three compounds are in R1, R2, R3, and R4. Among them, R1 mainly formed hydrogen bonds through residue binding, R2 formed hydrogen bonds and electrostatic interactions, and R3 and R4 could form hydrogen bonds and hydrophobic interactions with residues, respectively. From the analysis of the binding situation of the three molecules, for example, at the R1 position, BCX-2798 and zanamivir both have oxygen substitution groups, which can form a strong binding effect, while oseltamivir does not, indicating that the contribution to the binding energy here is little. R2 and R4 exhibited good binding affinity, in which the functional groups at the R2 position of the three drugs differed but all contained amines, and all substituted functional groups were alkyl at the R4 position. It indicated that R2 and R4 both made great contributions, but there were few differences among the three drugs. However, at the R3 position, they are all oxygen-substituted long-chain fatty acid groups with good binding affinity. However, it is an alkoxy group in oseltamivir, while the others have hydroxyl groups, making it easy to form more hydrophobic interactions. This indicates that the contribution of this position is greater than that of R1 when the leading needs optimization for designing drugs. The side-chain amide and carboxyl groups of oseltamivir, along with the two controls, play a crucial role in facilitating complex binding. Various substituents (guanidine, azide, and amino) at the C4 position (R2) interact with crucial amino acids to facilitate intermolecular binding. As shown in Figure 3B, the guanidine group of zanamivir, the azide group of BCX-2798, and the amino group of oseltamivir are strongly bound to GLU549, GLN215, and GLU276 through hydrogen bonding or electrostatic interactions, respectively. In addition, as shown in Figure 2B, oseltamivir docks to neuraminidase (NA) at the amino acid residue that also binds to these key groups (amino, carboxyl, and amides). Thus, oseltamivir may be a good choice for anti-PIV3 treatment with good docking results.

### Network proximity analysis of the candidates to PIV3

To further explore the potential candidates, we applied network proximity analysis to confirm it. A total of 324 disease targets were identified, with 314 targets prominently located in the PPI, to form the PIV3 disease module (*Z* = 18.71, *P*< 0.0001, randomly 1,000 times). In the PIV3 disease module, 231 targets formed the core disease module, and a total of 223 targets with *Z* = 6.66 and *p-value* = 3.6 × 10^-10^ formed the largest connected component (LCC), as shown in Supplementary Figure S2. It indicates that the localization of the LCC is significant in this disease module, which guarantees the formed PIV3 module is of good quality. Hence, network proximity analysis was based on this PIV3 disease module.

The targets of these 14 drugs were collected from the DrugBank and DGIdb databases to calculate network proximity (see Supplementary Figure S4). Then, the network proximity of the 14 candidates to the PIV3 disease module was calculated separately, and the results are also listed in Table 1. Because BCX-2798 has not yet been approved for clinical use, the related targets have not yet been identified, and the drug targets of telbivudine and peramivir cannot be located in the PPI, only 12 candidates had *Z*_*dc*_ values. Table 1 shows only four drugs, streptomycin, amikacin, oseltamivir, and ribavirin, with lower *Z*_*dc*_ than that of zanamivir (the control, *Z*_*dc*_ = −0.93). It means that these four drugs including oseltamivir were better candidates for anti-PIV3 treatment according to the network proximity analysis.

### Molecular dynamics simulation analysis

**FIGURE 4:**
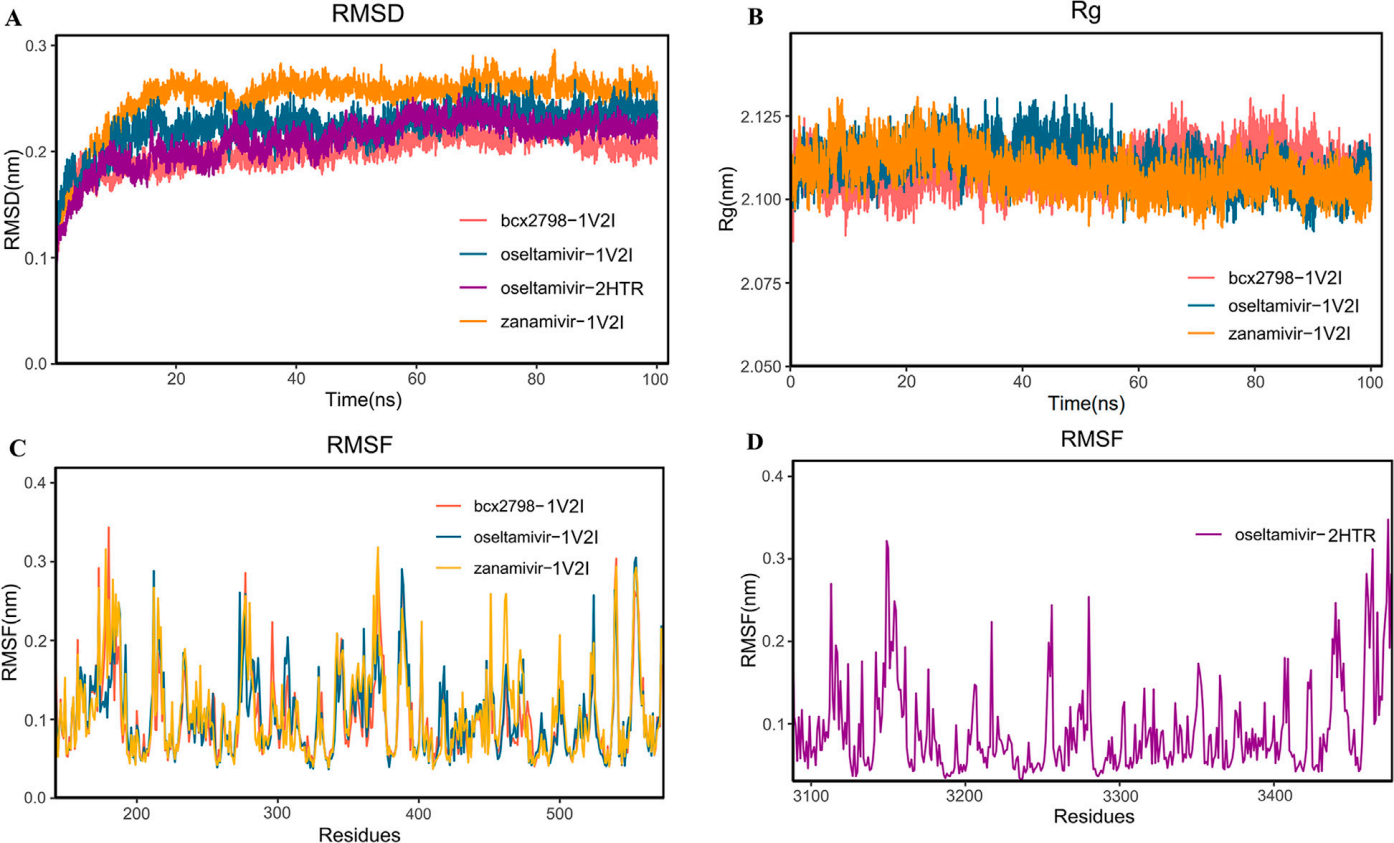
Molecular dynamics simulation analysis diagram of the complex at 100 ns. **(A)** *RMSD* analysis; **(B)** *RG* analysis; **(C, D)** *RMSF* analysis (red represents the bcx-2798–1V2I complex, yellow represents the zanamivir–1V2I complex, blue represents the oseltamivir–1V2I complex, and purple represents the oseltamivir–2HTR complex).

Combining the above three screening methods, we arrived at the results, of which only oseltamivir met all the aforementioned anti-PIV3 drug screening requirements. We used *RMSD* to assess the binding stability of the complexes formed by oseltamivir, zanamivir, and BCX-2798 with 1V2I over a period of 100 ns. As shown in Figure 4A, the *RMSD* of the oseltamivir–1V2I complex gradually stabilized after 40 ns, and the average *RMSD* of the oseltamivir–1V2I complex (0.22 nm) was significantly lower than that of the zanamivir–1V2I complex (0.25 nm) in the control group, indicating that it exhibited better structural stability. All complexes including oseltamivir–1V2I demonstrate good structural stability.

To assess the compactness of the complex structure, the *RG* values were also calculated for the three above complexes and listed in Figure 4B. As shown in Figure 4B, each set of complexes remained significantly compact throughout the 100-ns simulation. The average *RGs* of the oseltamivir–1V2I complex and the two control complexes were the same, 2.11 nm. The oseltamivir–1V2I complex was similar to the two control complexes with a smaller *RG* in higher density, which led to a more stable system structure. As shown in Figure 4C, the *RMSF* illustrates the flexibility of the molecule (Awan et al., 2024), and the average *RMSF* of zanamivir is 0.11 nm, while the mean *RMSFs* of both BCX-2798 and oseltamivir were 0.10 nm. Furthermore, in Figure 4C, the three complexes show similar fluctuations in residue levels in different regions, leading to improved conformational optimization during substrate binding. Lower *RMSF* values may indicate active sites or binding sites, as these regions tend to become more stable after binding ligands. In oseltamivir–HN, the *RMSF* values of the key residues GLU276 (0.12 nm), GLU409 (0.13 nm), ARG424 (0.08 nm), THR426 (0.10 nm), and ARG502 (0.10 nm) at the binding pocket were very close to the average *RMSF* (0.10 nm), indicating that the binding between oseltamivir and the protein was relatively stable. Both the *RMSD* and *RG* of oseltamivir–HN indicate that the system is stable. The *RMSF* also shows stable residues near the ligand binding site, leading us to consider the complex binding to be stable.

Simultaneously, the binding free energies of the three complexes were calculated by using MM/PBSA and MM/GBSA, thereby determining the ligands’ binding affinity at the protein active site (Bashir et al., 2023), and the results are shown in Table 2. The binding energy values obtained from the two algorithms are quite similar. The total binding free energies of oseltamivir–1V2I (−10.60 kal/mol) and the two controls were less than −9.55 kal/mol (normal threshold for strong binding), indicating strong binding ability for both compounds. Therefore, oseltamivir was a good candidate according to results of molecular dynamics simulation.

**TABLE 2:** Binding energies of three complexes (kcal/mol).

Parameters	Zanamivir	BCX-2798	Oseltamivir
MMPBSA			
$∆G_{vdw}$	−32.95	−12.65	−20.70
$∆G_{ele}$	−144.42	−83.30	−20.87
$∆G_{P}$	163.58	86.40	35.16
$∆G_{NP}$	−3.63	−1.98	−4.19
∆Ggas	−177.37	−95.95	−41.57
∆Gsol	159.95	84.43	30.97
∆G TOTAL	−17.42	−11.52	−10.60
MMGBSA			
$∆G_{vdw}$	−33.17	−14.76	−21.42
$∆G_{ele}$	−139.74	−93.21	−13.80
$∆G_{P}$	154.27	94.82	24.82
$∆G_{NP}$	−4.56	−2.55	−4.60
∆Ggas	−172.92	−107.97	−35.22
∆Gsol	149.70	92.27	20.22
∆G TOTAL	−23.21	−15.71	−15.00

## Discussion

In this article, we explored an integrated drug repurposing method, including disease similarity and chemical similarity as multi-similarity analysis approaches, molecular docking and molecular dynamic simulation methods as structure-based screening approaches, and network proximity analysis, in which we quantified the network distance between the disease module of PIV3 and the drug targets to probe the potential anti-PIV3 drugs. In addition, according to this drug repurposing protocol, we confirm that oseltamivir is the best potential anti-PIV3 drug.

In our research results, a similar pattern was observed when oseltamivir inhibited PIV3 and influenza A. As is known, zanamivir is a successful structural design drug for NA inhibitor, and oseltamivir is designed using zanamivir as the lead compound. Among them, the side-chain amides, carboxyl groups, and amino groups play a crucial role in inhibiting the influenza A virus (Laborda et al., 2016; Tao et al., 2022), and the acetamido fragment can be well-accommodated within the cavity-binding domain (Guillon et al., 2014). In our study, oseltamivir showed good chemical similarity to zanamivir and BCX-2798. Their shared side-chain amide and carboxyl group result in similar interactions when bound to HN using molecular docking, respectively. HN can accommodate larger acyl groups in the C5 binding region (Guillon et al., 2014), and the amides of all three complexes provide hydrogen bonds for protein binding. In the oseltamivir–HN complex, the amide also forms hydrogen bonds with THR426, which greatly contributes to the stable binding of the complex. This advantage distinguishes it from other complexes. The carboxylic acid side chain of oseltamivir forms hydrogen bonds with the surrounding arginine (ARG192 and ARG424), which enhances the binding stability between molecules.

Though some unique structures of oseltamivir can also facilitate its binding to HN, various substituents at position C4 (e.g., guanidine, azido, amide, and amino group) can enter the cavity around the residue of the active site of PIV3, thereby inhibiting the activity of PIV3 (El-Deeb et al., 2014; Rota et al., 2023). Specifically, the guanidine group of zanamivir is tightly bound to the hydrogen bond of GLU549, and the azido group of BCX-2798 can form hydrogen bonds with GLN215. The addition of the azido group significantly inhibits the activity of PIV (Chibanga et al., 2019). The amino group at the C4 position of oseltamivir enhances the electron-donating capability of nitrogen ions. This enhancement allows for the formation of an electrostatic attraction with GLU276 and GLU409, thereby increasing the charge attraction and binding force between molecules. Esterification of the oseltamivir side-chain carboxyl group leads to the elongation of the backbone, enabling it to penetrate deeper into the grooves of the binding site, thereby increasing the tightness of the binding complex. Therefore, the utilization of the chemical similarity method can offer more effective information for the development of anti-PIV3 drugs. Additionally, employing molecular docking in conjunction with molecular dynamics simulation methods can enhance the exploration of the structural interactions.

Searching for drugs based solely on chemical structure makes it difficult to consider the genetic regulatory processes of the disease itself. The synergistic functions of a large number of genes during disease initiation and progression were considered in this study. The association between PIV3 and oseltamivir based on genes was explored using network proximity analysis. The proximity can also be used to define the similarity between two drug candidates and covered several drug–disease associations. If a drug is proximal to the PIV3 disease, it is more likely to be effective than a distant drug. It indicated that the oseltamivir targets are more closely linked to the PIV3 disease module than those of the control (zanamivir). The network proximity of candidate targets to the PIV3 disease genes provides special insights into the candidate mechanism of action, uncovering the patho-biological components targeted by candidates, and improves the feasibility and interpretability for drug repurposing.

However, if some drugs cannot be localized within the PPI network due to the incomplete human interactome network, these drugs may not be screened by network proximity. Repurposed drugs may skip phase-I clinical trials, but they still need to undergo phase-II and phase-III trials to assess their efficacy against new diseases. Although there are some limitations to this study, this approach allows for the rapid identification of potential therapeutic agents to mitigate the effects of emerging epidemics.

While computational methods are robust, clinical data or experimental validation of oseltamivir’s inhibitory effect on PIV3 would significantly strengthen the findings. The clinical data were collected from the Second Affiliated Hospital of Chongqing Medical University, the Third Affiliated Hospital of Chongqing Medical University, and the Children’s Hospital of Chongqing Medical University by searching on Yidu Cloud (https://www.yiducloud.com.cn/, from January 2016 to June 2024). There were 539 PIV3 cases, including 479 cases of children and infants under 6 years of age, 14 cases of individuals aged 7–17 years, 18 cases of individuals over 60 years old, and only 28 adult cases. Till now, there were no clinically approved antiviral drugs for treating PIV3 infection. Among 539 PIV3 patients, most mainly received expectorant cough medicine and nebulizer therapy without antiviral treatment, 203 patients received ribavirin treatment, and 17 patients were treated with oseltamivir. Of those 17 patients who were administered off-label oseltamivir, the patients did not undergo further treatment according to the medical records. This suggests that oseltamivir has a certain efficacy in treating PIV3 in clinical settings, aligning with the findings of our result. Given that most PIV3-infected patients are children, ribavirin can lead to severe adverse clinical reactions such as hemolytic anemia and teratogenic mutations (Sinclair et al., 2017). Oseltamivir is deemed safe for children over 1 year of age as an influenza treatment and has been FDA-approved for infants and young children over 14 days old for enhanced safety (Malosh et al., 2018). Therefore, we recommend considering oseltamivir for clinical application.

## Conclusion

Oseltamivir is screened as a potential anti-PIV3 drug through a variety of drug repurposing methods as it has been considered an effective drug in clinical off-label medications for anti-PIV3 treatment in some children’s hospitals in recent years. Oseltamivir exhibited high similarity to potential PIV3 inhibitors (zanamivir and BCX-2798), demonstrated strong binding ability to the key target protein HN, and was closely related to the PIV3 disease module. Therefore, oseltamivir fulfilled all the screening requirements and emerged as the most effective anti-PIV3 drug. The molecular docking results revealed that oseltamivir and PIV3 were bound through hydrogen bonding, electrostatic attraction, and hydrophobic interactions. In particular, the amides, carboxyl, and amino groups in oseltamivir are important structures for inhibiting PIV3. This multi-similarity drug repurposing method will be a feasible reference for other disease and drug repurposing research.

## Data Availability

The original contributions presented in the study are included in the article/supplementary material, further inquiries can be directed to the corresponding author.
