# Supplementary material for: Drug repurposing to tackle parainfluenza 3 based on multi-similarities and network proximity analysis
Source: Front Pharmacol. 2024 Oct 1;15:1428925. doi: 10.3389/fphar.2024.1428925 (PMC11473393; doi:10.3389/fphar.2024.1428925)
Supplement: Supplementary file 2 [file DataSheet1.docx]

**Drug Repurposing to Tackle Parainfluenza 3 Based on Multi-similarities and Network Proximity Analysis**

Xinyue Chen ^1^, Bo Zhou ^1,2^, Xinyi Jiang ^1^, Huayu Zhong ^1^, Aijing You ^3^, Taiyan Zou ^1,4,5^, Chengcheng Zhou ^1^, Xiaoxiao Liu ^1^, Yonghong Zhang ^1,4,5*^

^1^ Chongqing Key Research Laboratory for Drug Metabolism, College of Pharmacy, Chongqing Medical University, Chongqing, China

^2^ Department of Pharmacy, Children’s Hospital of Chongqing Medical University, Chongqing, China.

^3^ The Second Clinical College of Chongqing Medical University, Chongqing, China

^4^ Medical Data Science Academy, College of Medical Informatics, Chongqing Medical University, Chongqing, China

^5^ Chongqing Engineering Research Center for Clinical Big-data and Drug Evaluation, Chongqing Medical University, Chongqing, China

***Corresponding author:**

Yonghong Zhang, Email: [zhyvonne26@cqmu.edu.cn](mailto:zhyvonne26@cqmu.edu.cn) ; Tel: +86 13896191038.

**Supporting Information**

Total pages of this word document: 8

Total supporting figures: 2

Total supporting tables: 4

There are 2 supporting figure (Figure S1-S2) and 4 supplementary tables (Table S1-S4) in this Supporting Information. Two supporting figures are listed in this document, while other 4 supplementary tables are given as 4 excel documents, separately.

**Supporting figures captions**

Figure S1 Chemical structures of 12 drugs obtained by preliminary screening

Figure S2 The statistic results of the localization of the largest connected component in disease module

| 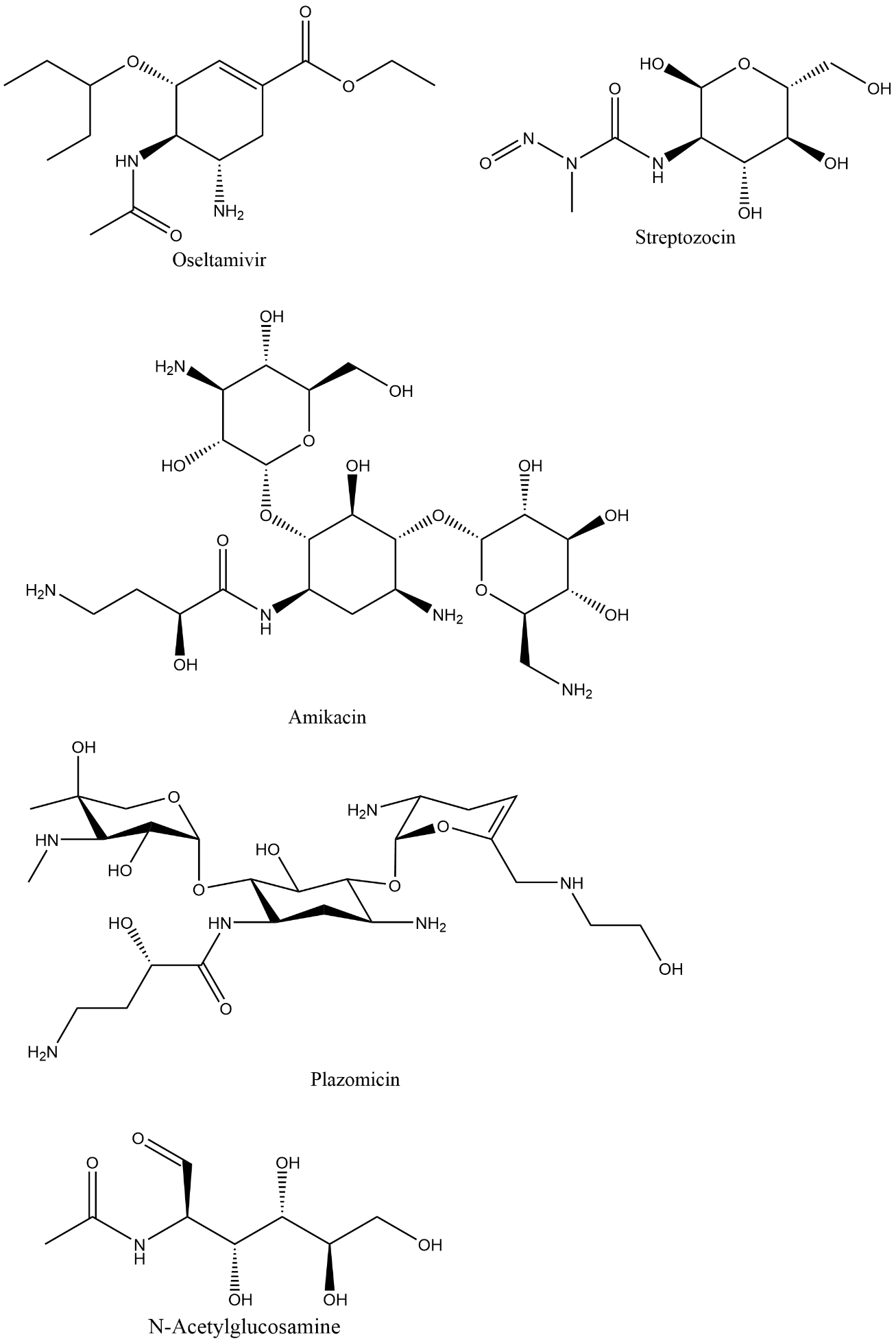  A | 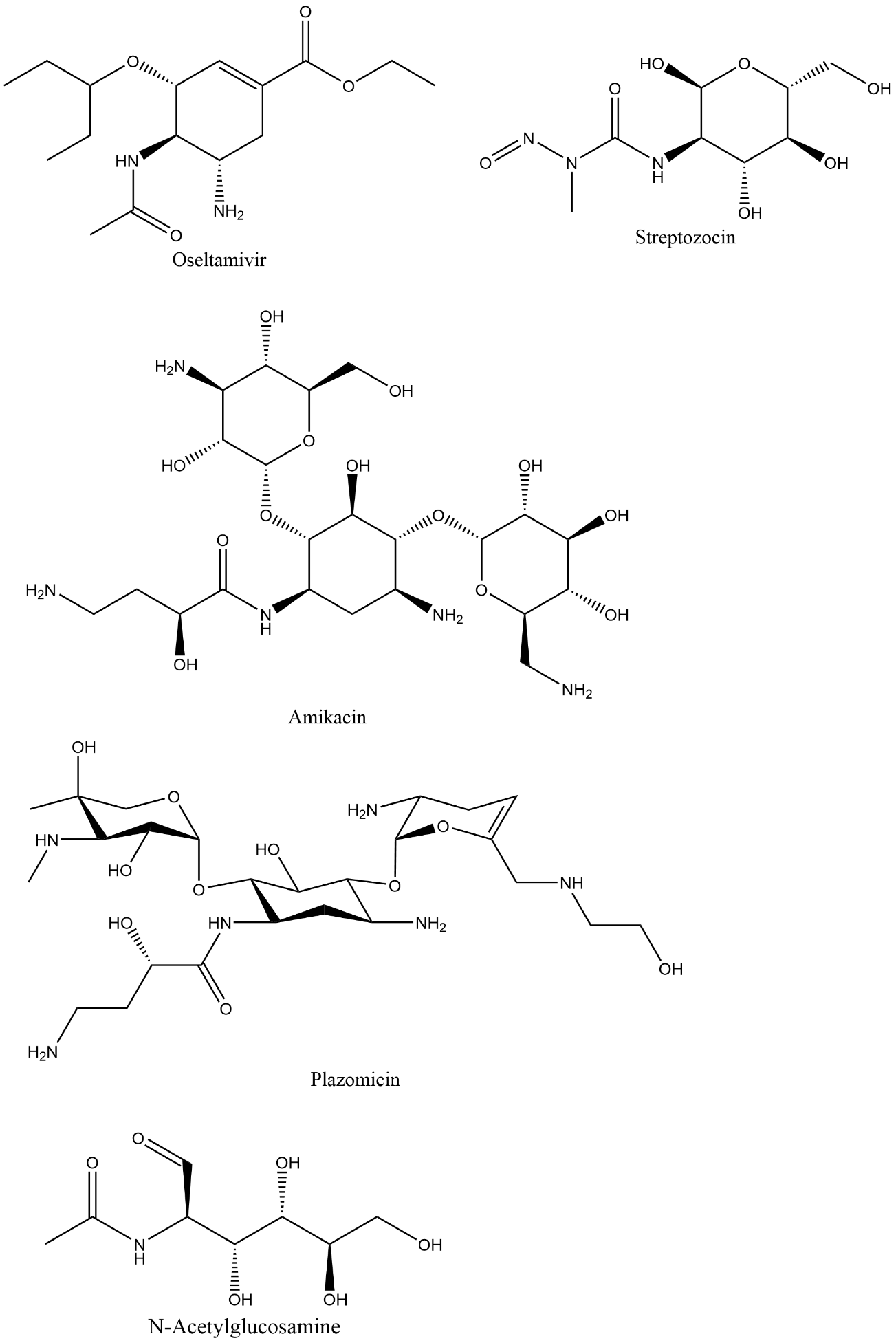  B |
| --- | --- |
| 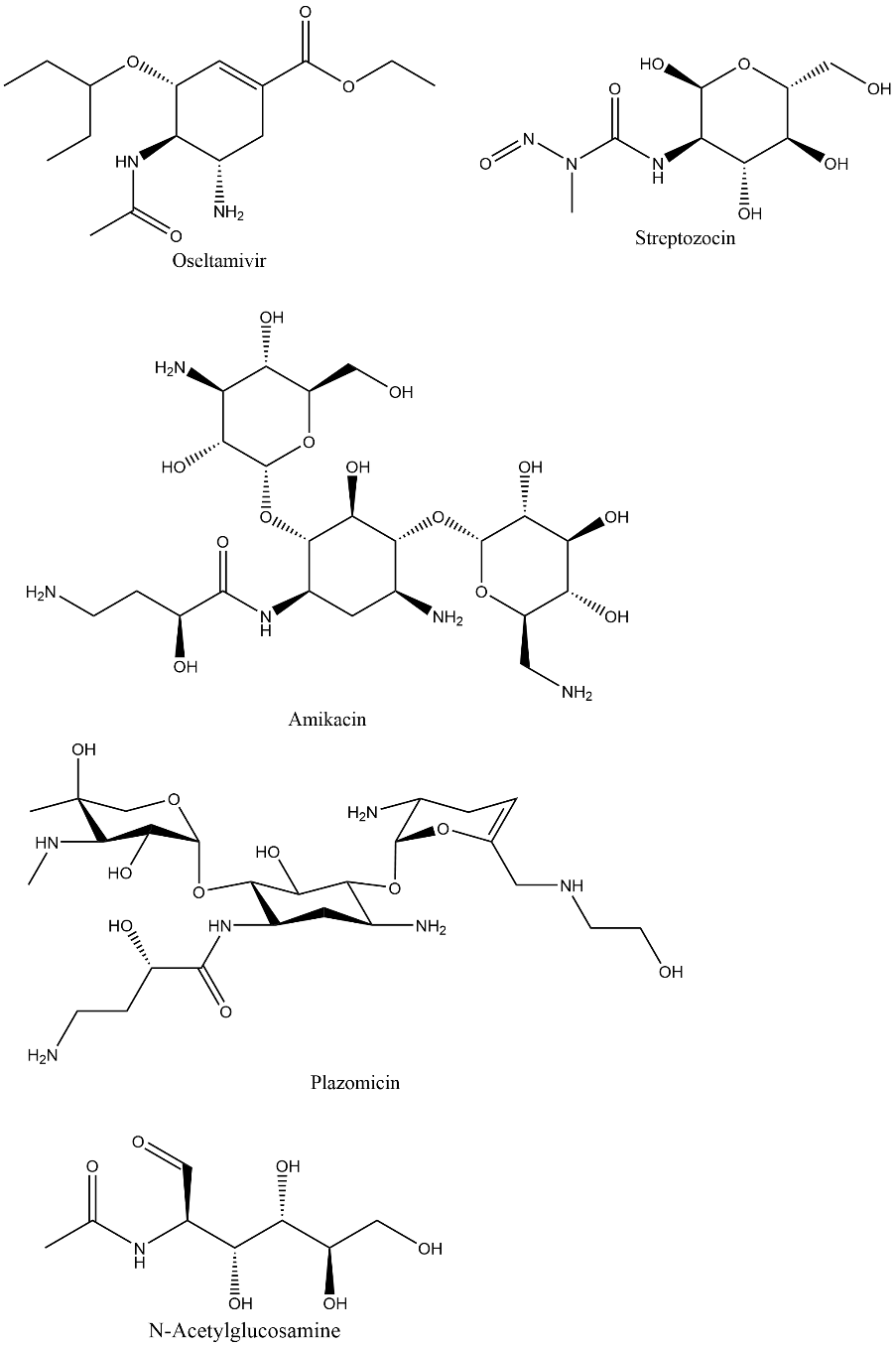  C | 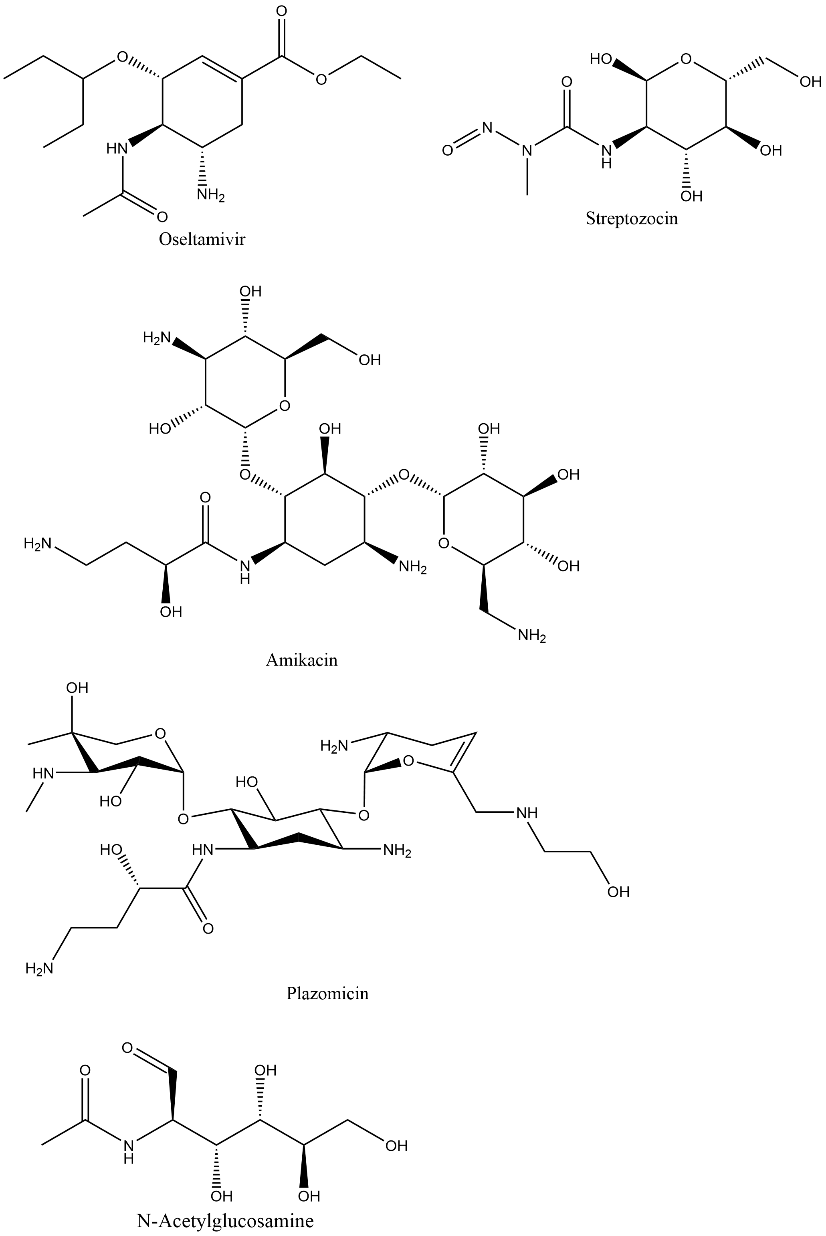  D |
| 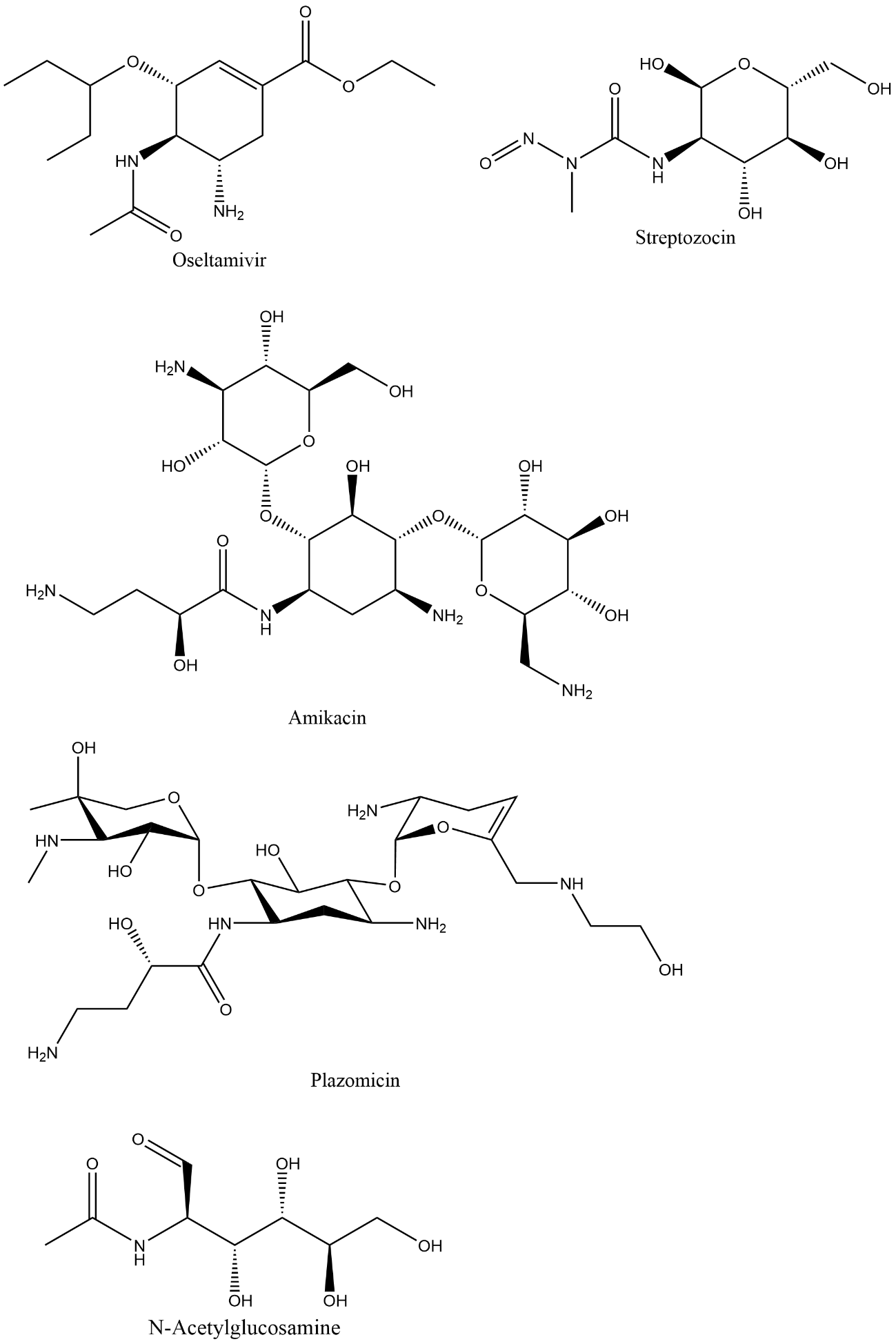  E | 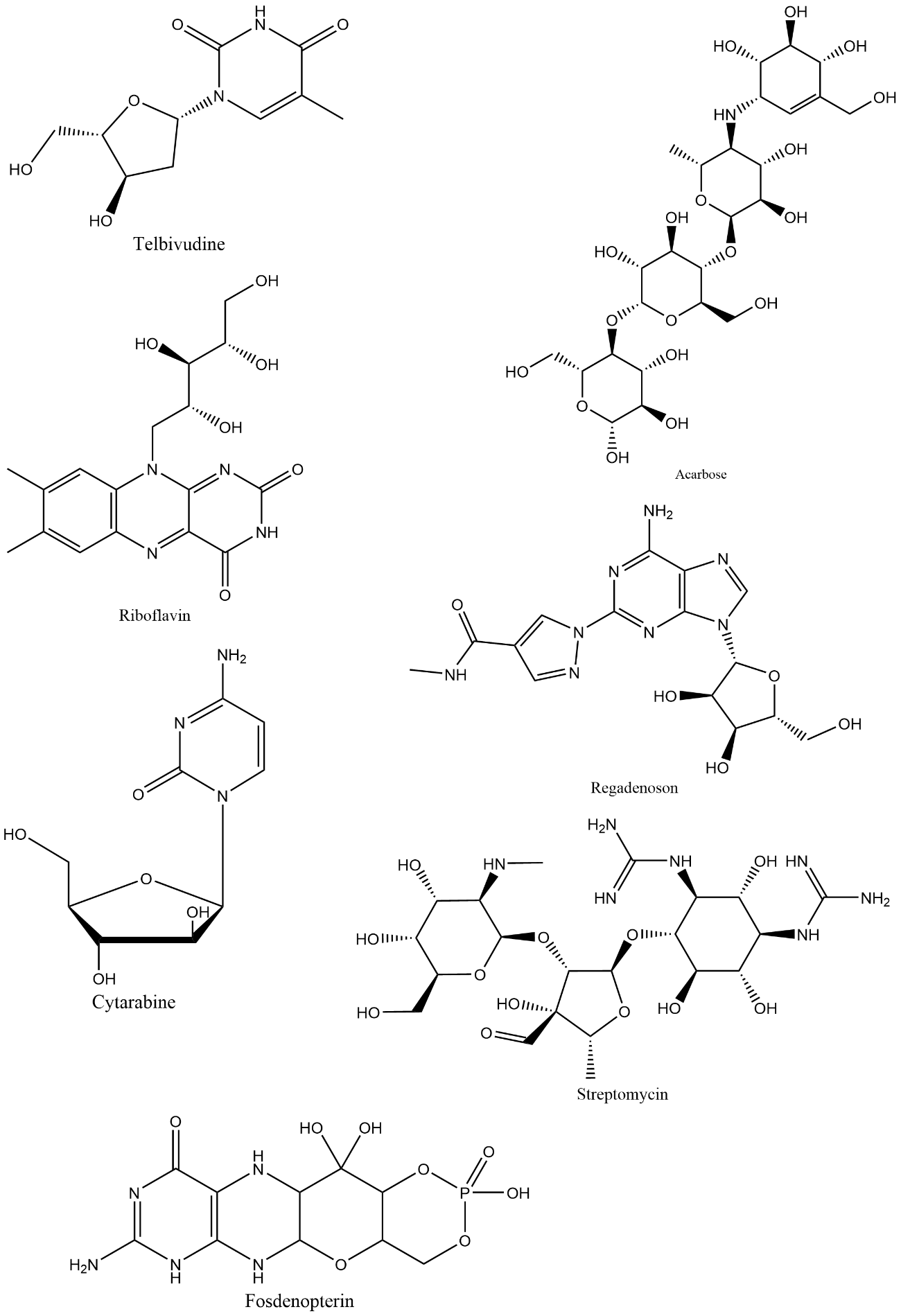  F |
| 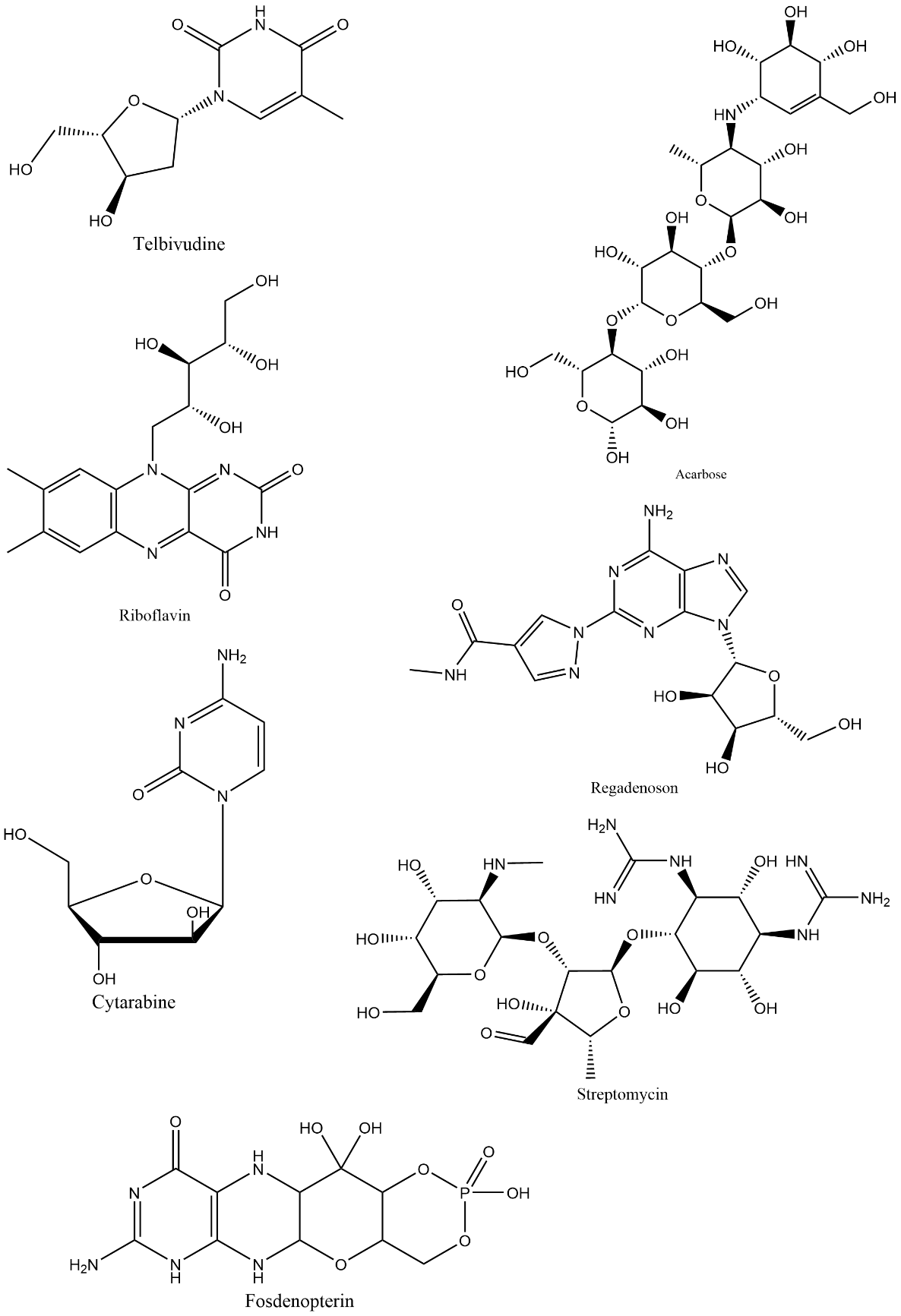  G | 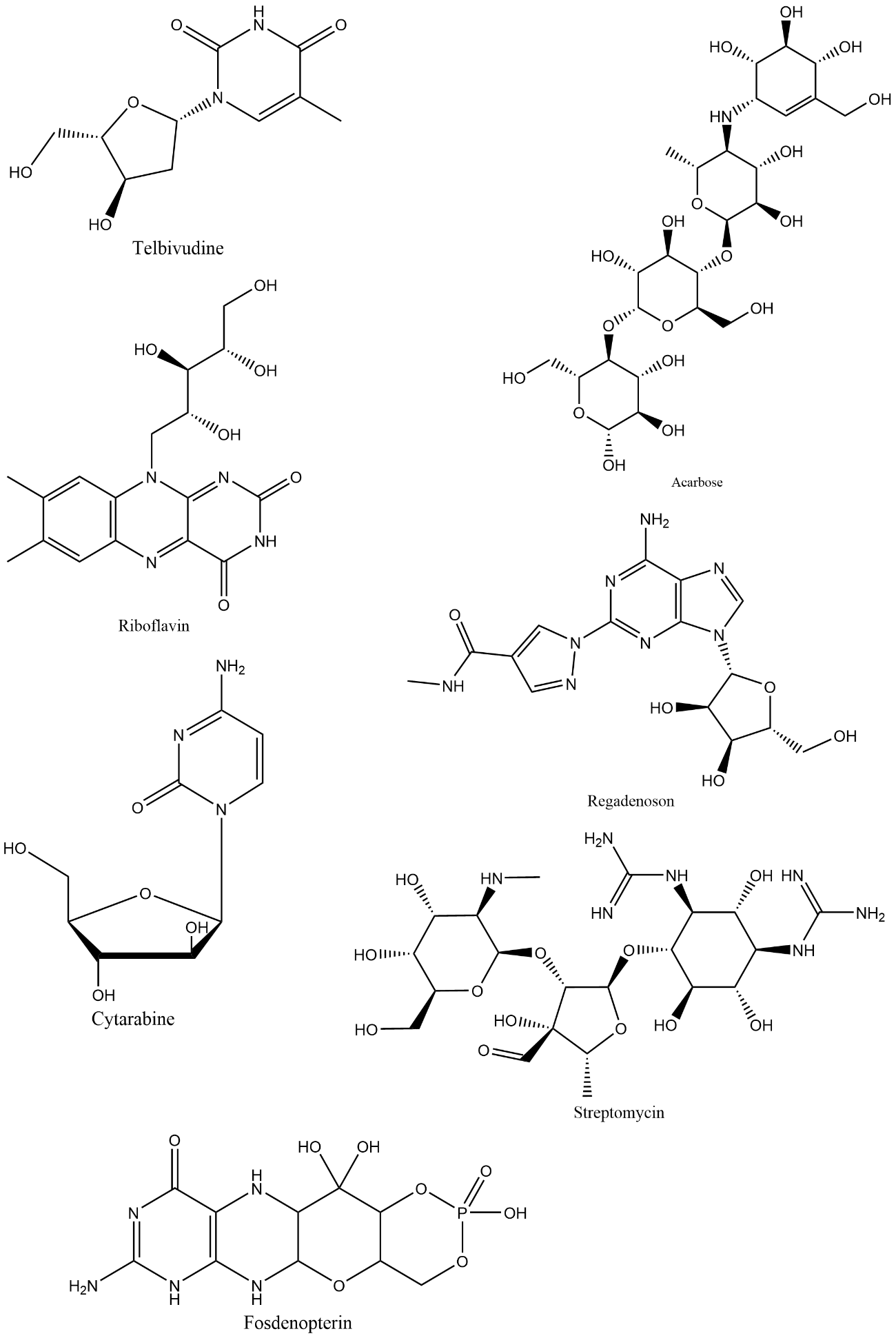  H |
| 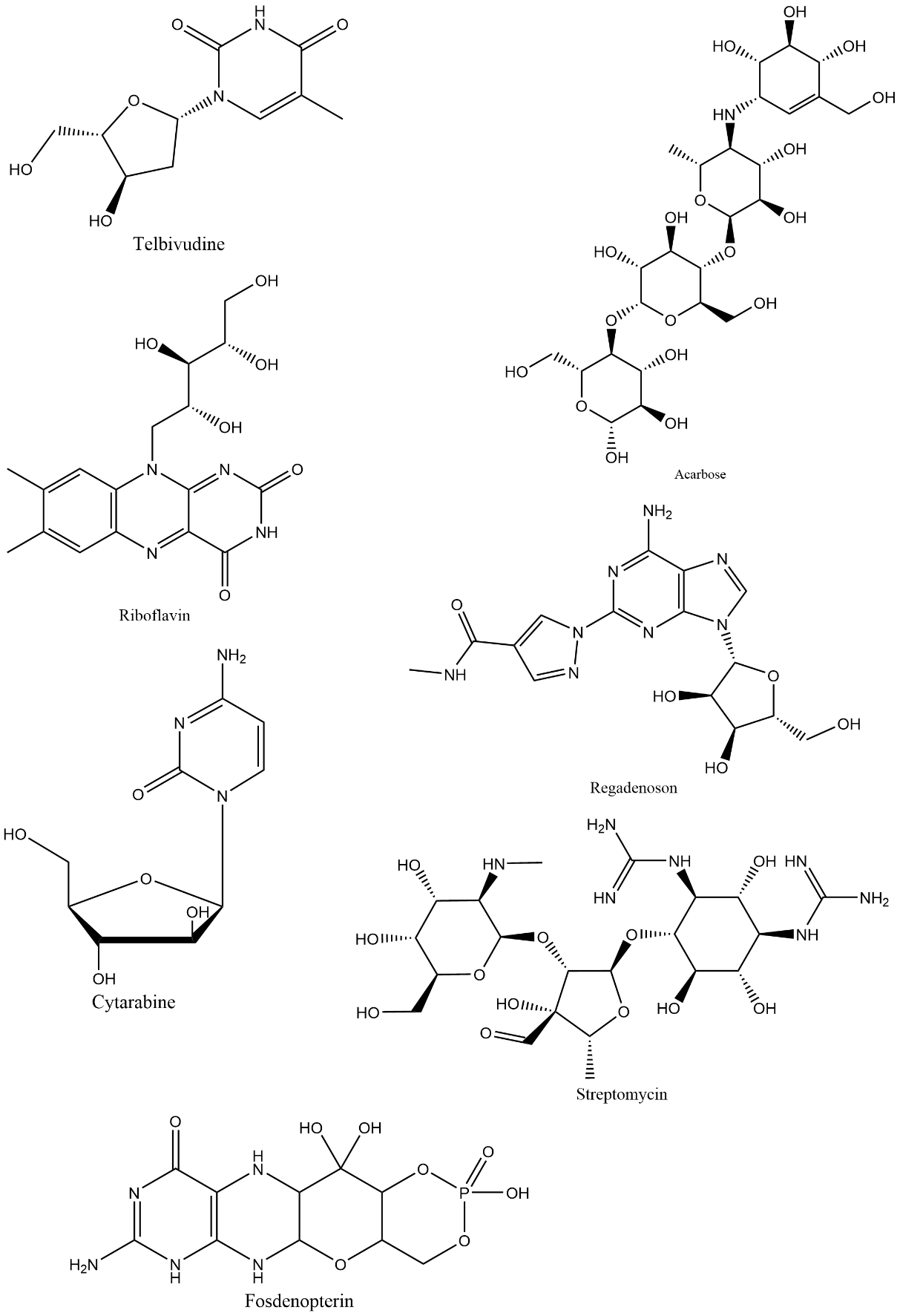  I | 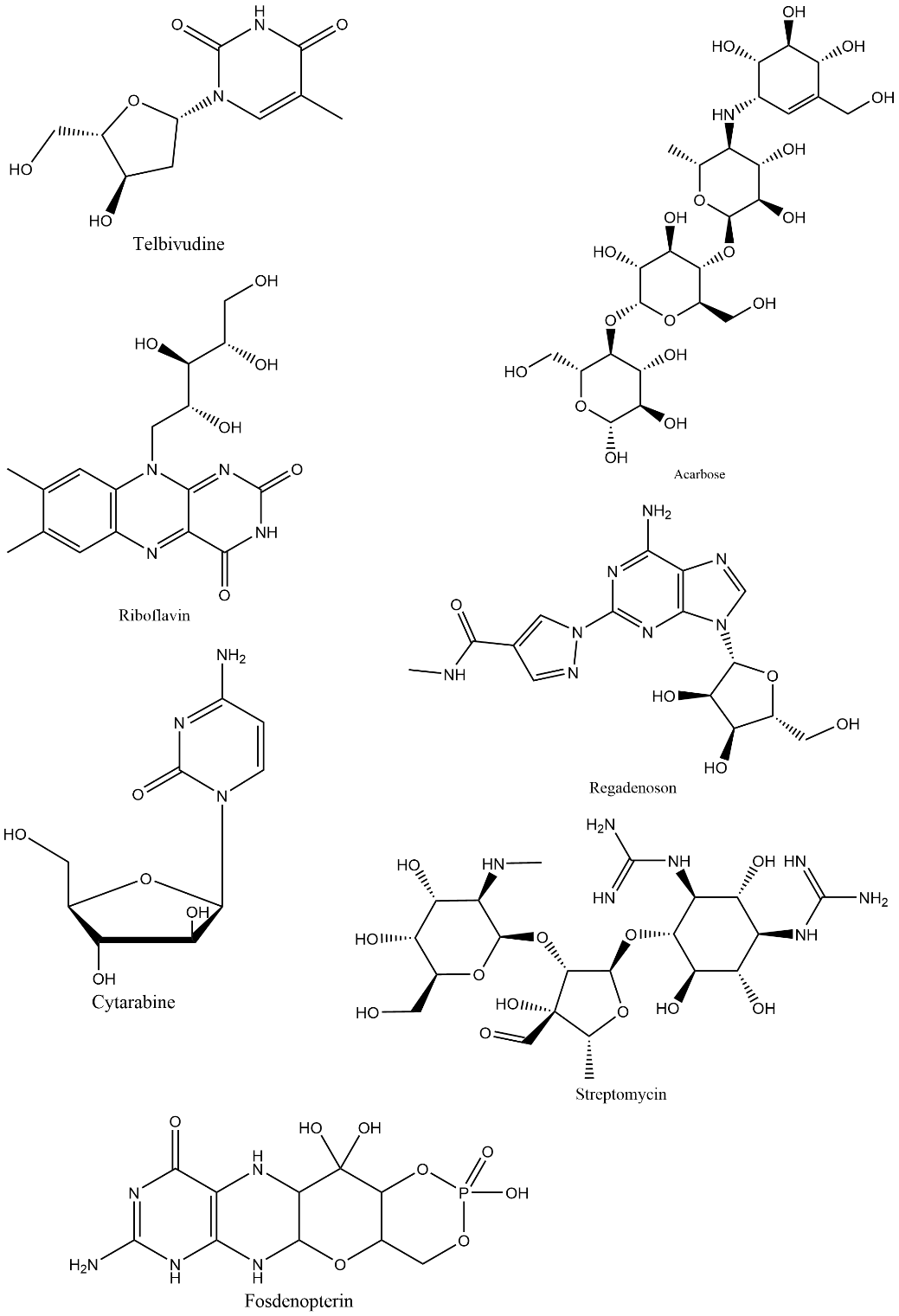  J |
| 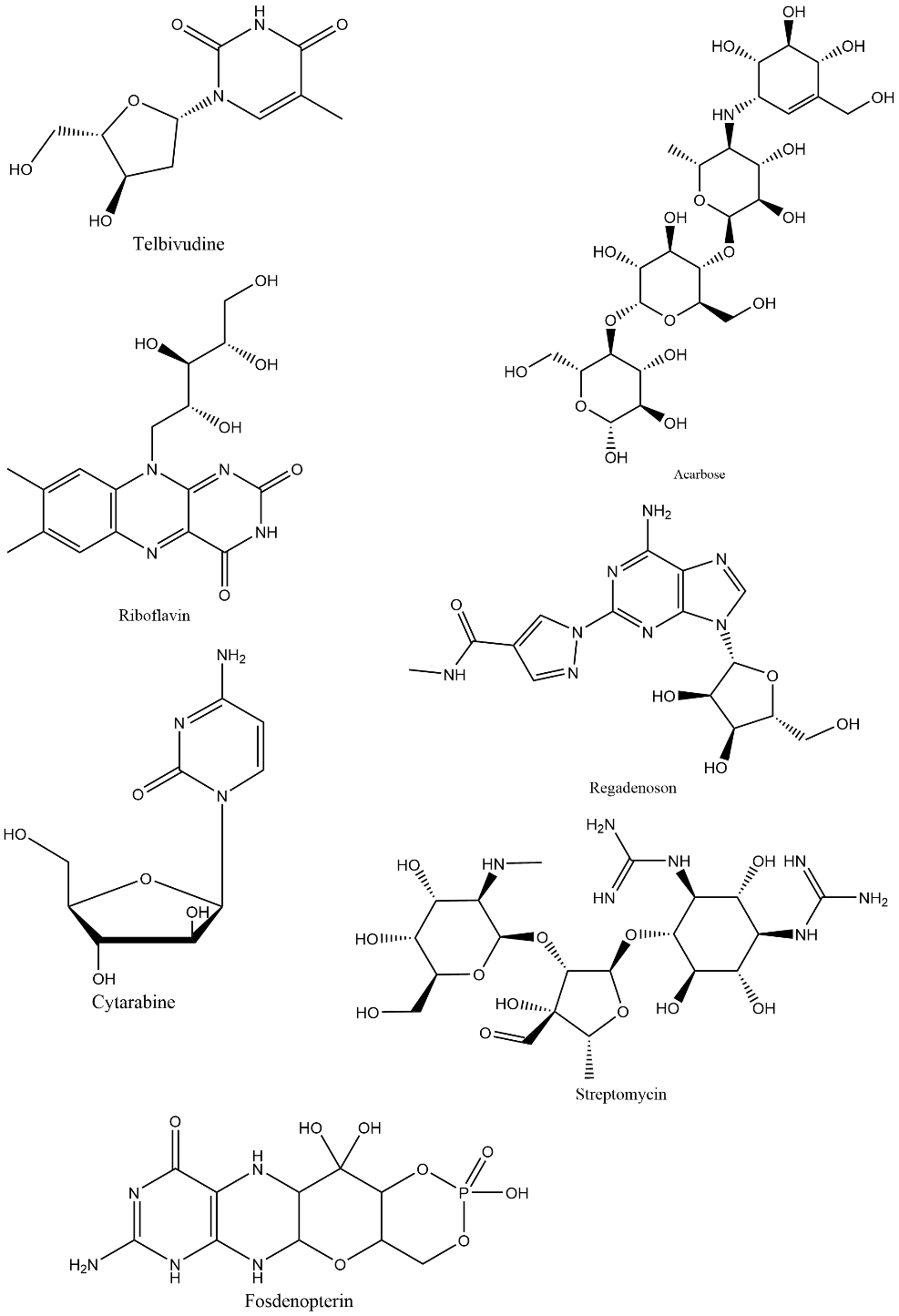  K | 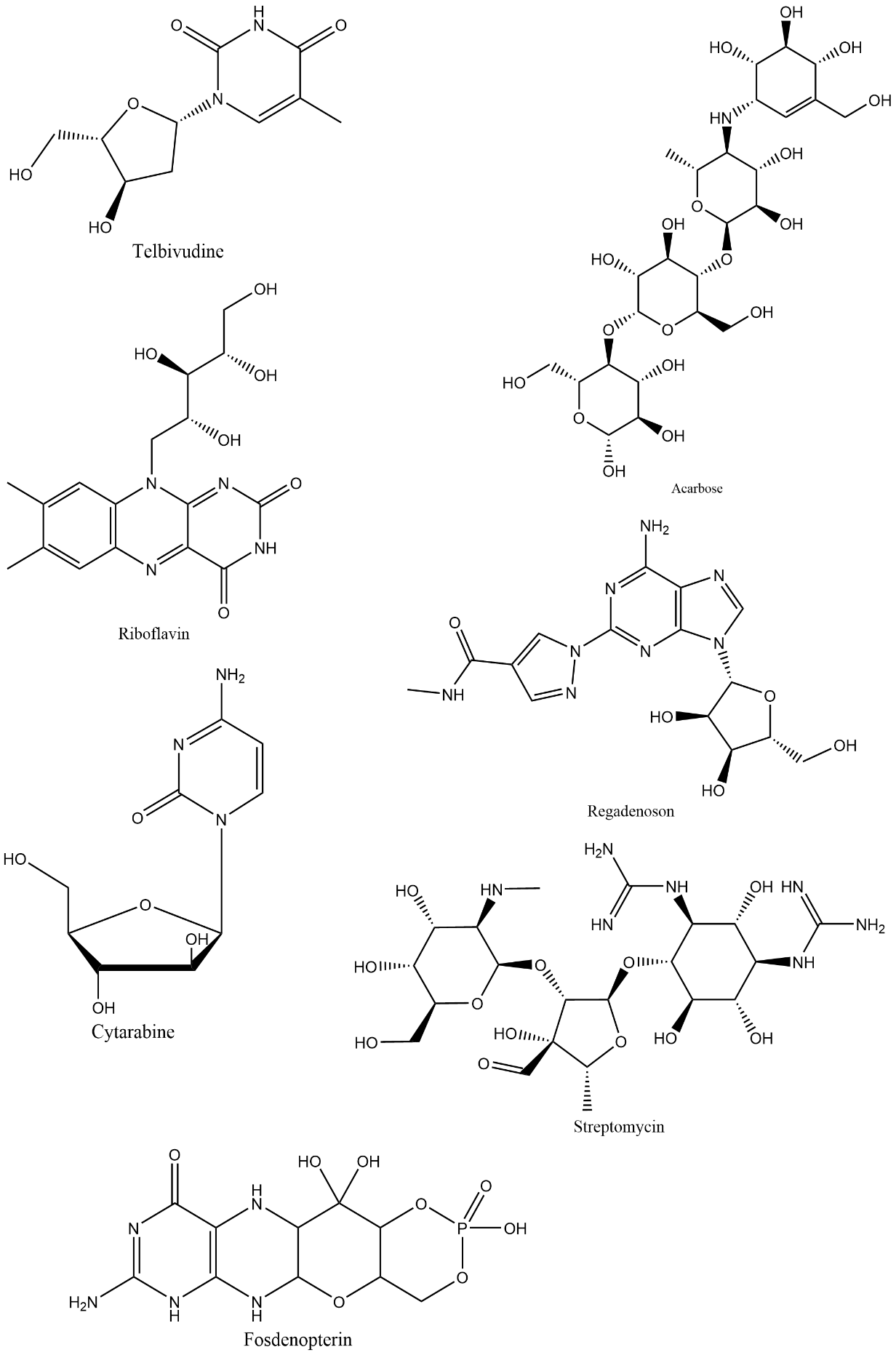  L |
| 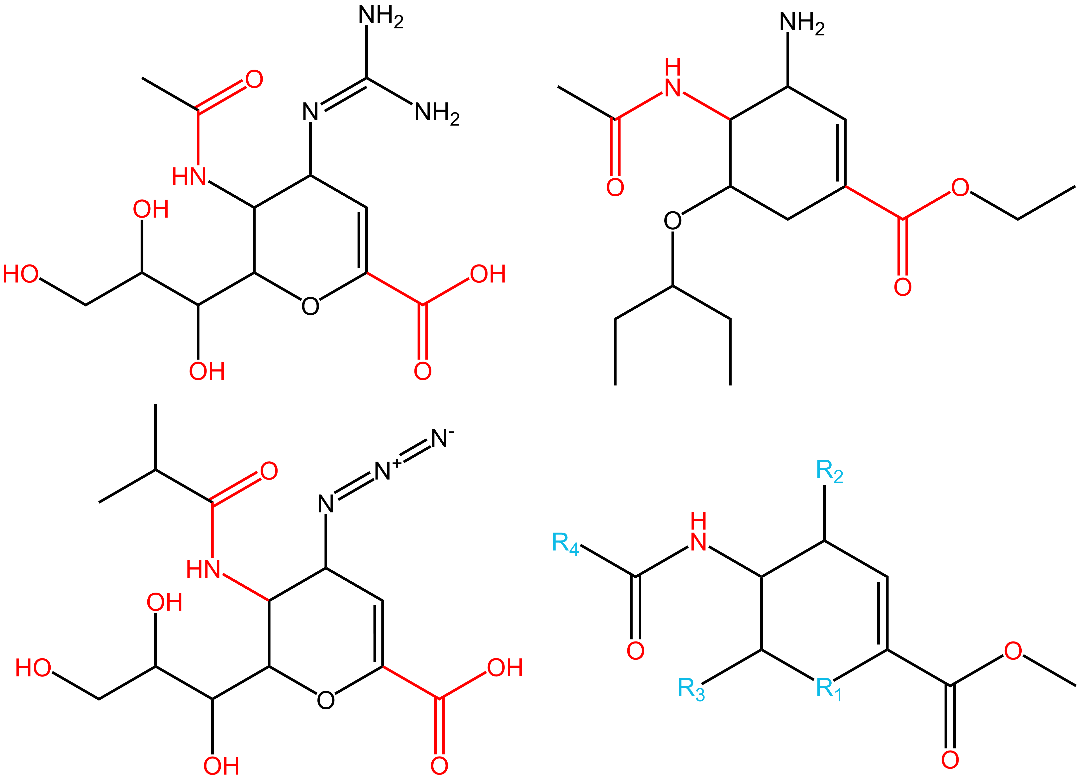  M | 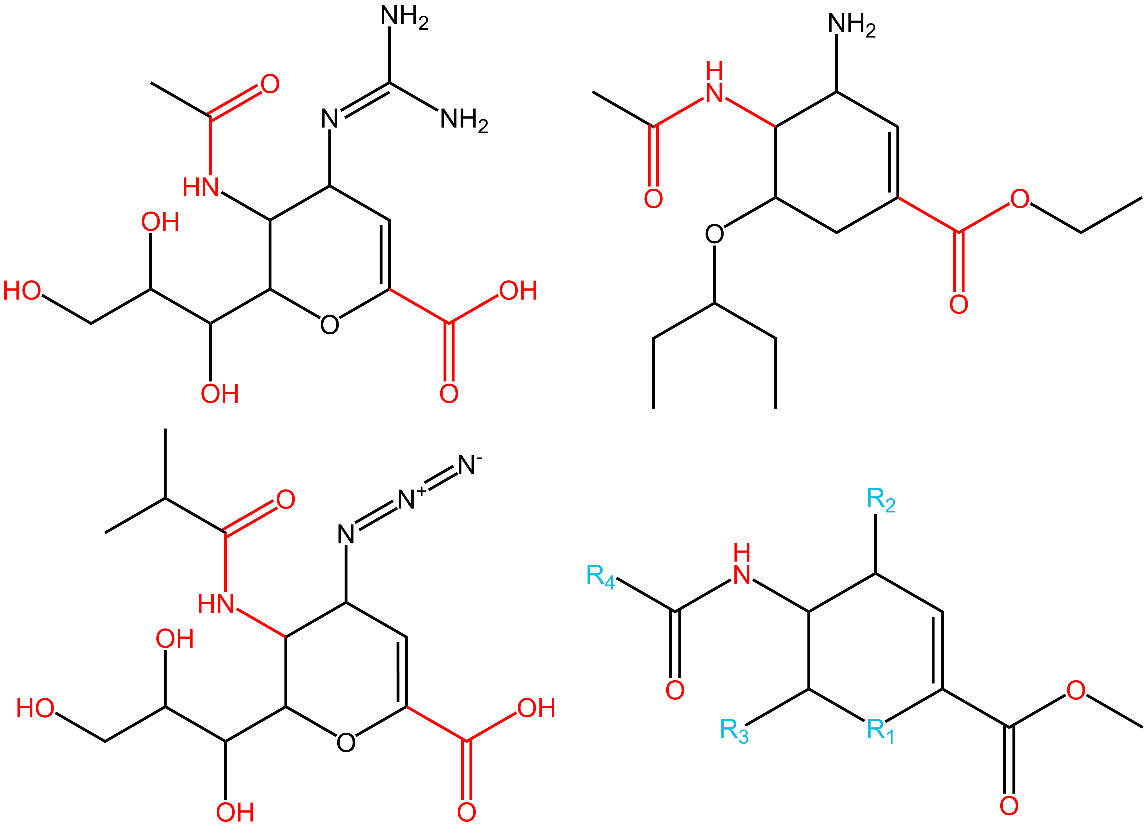  N |

Figure S1 Chemical structures of 12 drugs obtained by preliminary screening(A is Oseltamivir，B is Streptozocin，C is Amikacin，D is Plazomicin，E is N-Acetylglucosamine，F is Telbivudine, G is Acarbose，H is Riboflavin，I is Regadenoson，J is Streptomycin，K is Cytarabine，L is Fosdenopterin, M,N are controls zanamivir and BCX-2798).

| 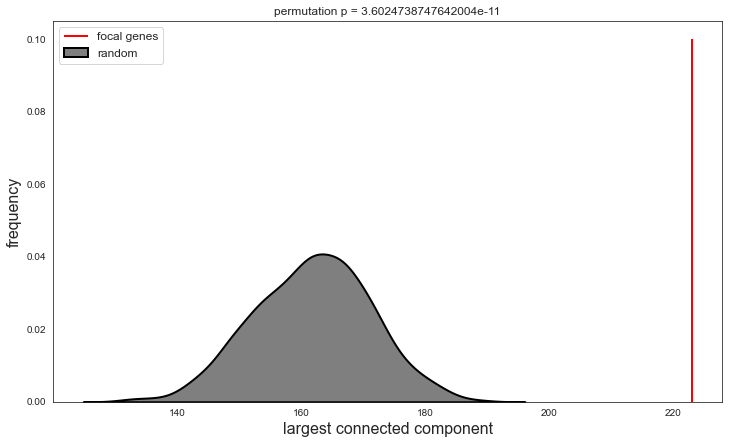 |
| --- |

Figure S2 The statistic results of the localization of the largest connected component in disease module. (The grey part is the statistic results of Prand (*S*) and *focal genes* represent the frequency of 314 disease genes in our PIV3 disease module P (*S*). It indicates the localization of the largest connected component in disease module is significant.)

**List of supporting tables**

Table S1 Chemical Similarity of Zanamivir, BCX2798 and 2585 Approved Drugs. (A) The SMILES structures of 2585 approved drugs (B) Similarity to zanamivir (C) Similarity to BCX-2798

Table S2 324 PIV3 targets

Table S3 The disease similarity of PIV3 to 1364 diseases(A) The MeSH ID for 1364 diseases (B) Disease similarity to PIV3.

Table S4 Drug targets for 14 drugs
